# Natural products for kidney disease treatment: Focus on targeting mitochondrial dysfunction

**DOI:** 10.3389/fphar.2023.1142001

**Published:** 2023-03-15

**Authors:** Jiewu Huang, Ye Liang, Lili Zhou

**Affiliations:** State Key Laboratory of Organ Failure Research, Guangdong Provincial Key Laboratory of Renal Failure Research, National Clinical Research Center for Kidney Disease, Guangdong Provincial Institute of Nephrology, Division of Nephrology, Nanfang Hospital, Southern Medical University, Guangzhou, China

**Keywords:** natural products, mitochondrial dysfunction, energy metabolism, kidney diseases, mitochondrial homeostasis

## Abstract

The patients with kidney diseases are increasing rapidly all over the world. With the rich abundance of mitochondria, kidney is an organ with a high consumption of energy. Hence, renal failure is highly correlated with the breakup of mitochondrial homeostasis. However, the potential drugs targeting mitochondrial dysfunction are still in mystery. The natural products have the superiorities to explore the potential drugs regulating energy metabolism. However, their roles in targeting mitochondrial dysfunction in kidney diseases have not been extensively reviewed. Herein, we reviewed a series of natural products targeting mitochondrial oxidative stress, mitochondrial biogenesis, mitophagy, and mitochondrial dynamics. We found lots of them with great medicinal values in kidney disease. Our review provides a wide prospect for seeking the effective drugs targeting kidney diseases.

## 1 Introduction

Kidney diseases, including acute kidney injury (AKI) and chronic kidney diseases (CKD), are becoming a public health problem. The prevalence of kidney diseases is over 10% worldwide (Eckardt et al., 2013; Glassock et al., 2017; Huang et al., 2021). Notably, a large majority of CKD patients would inevitably progress into end-stage renal diseases (ESRD), a diseased state depending on dialysis or transplantation to survive. These treatments result into the heavy financial and medical burdens (Levey and Coresh, 2012). However, even on dialysis or transplantation, kidney diseases are still facing the high mortality rates (Vijayan, 2021).

With a massive mass of mitochondria (Forbes and Thorburn, 2018; Zhang et al., 2021a), the prominent organelle for adenosine triphosphate (ATP) production (Forbes and Thorburn, 2018), kidney is the second highest organ consuming oxygen at resting state. Tubular epithelial cells (TECs) are the main constituent in kidney parenchyma. They, on huge demand of ATP supply, undertake active reabsorption and secretion (Forbes and Thorburn, 2018). Other renal cells, like podocytes, also require a high supply of energy. In physiological condition in kidney, the most proportion of ATP is from oxidative phosphorylation (OXPHOS) in mitochondrial matrix. Mitochondrial homeostasis is vital to maintain normal kidney function (Forbes and Thorburn, 2018), as mitochondrial dysfunction not only contributes to energy deficiency, but also leads to disruption of cellular homeostasis and renal function (Bhargava and Schnellmann, 2017). The main phenomenon of mitochondrial dysfunction is the excessive generation of reactive oxygen species (ROS), which plays a key role in cell injury and kidney diseases (Linkermann et al., 2014; Dan Dunn et al., 2015; Kang et al., 2015; Pickles et al., 2018; Zhao et al., 2021b; Liang et al., 2021). As reported, mitochondrial dysfunction contributes to nearly all kinds of kidney diseases, such as diabetic kidney diseases (DKD) (Tan et al., 2021), inherited renal diseases (Emma et al., 2016), podocytopathy (Arif et al., 2019), IgA nephritis (Yu et al., 2019), hypertensive nephritis (Eirin et al., 2015) and so on.

Natural products are defined as the mixture and monomer from natural sources of animals, plants and microorganisms. They have exhibited a wide variety of biological activities for thousands of years, including targeting mitochondrial dysfunction (Katz and Baltz, 2016; Mu et al., 2020; Liang et al., 2021). Large reports have shown natural products have strong therapeutic values in promoting mitochondrial biogenetics and energetics, reducing mitochondrial ROS, enhancing mitophagy, and regulating mitochondrial dynamics (Mu et al., 2020; Liang et al., 2021). Therefore, natural products may exert beneficial effects for protecting kidney. However, their therapeutic values in kidney diseases have not been extensively reviewed. In our review, we introduced natural products targeting mitochondrial dysfunction in kidney diseases in detailed comments. Our review provides the important potential strategies for clinical treatments.

## 2 Mitochondrial dysfunctions in kidney diseases

### 2.1 Mitochondrial homeostasis imbalance

**FIGURE 1:**
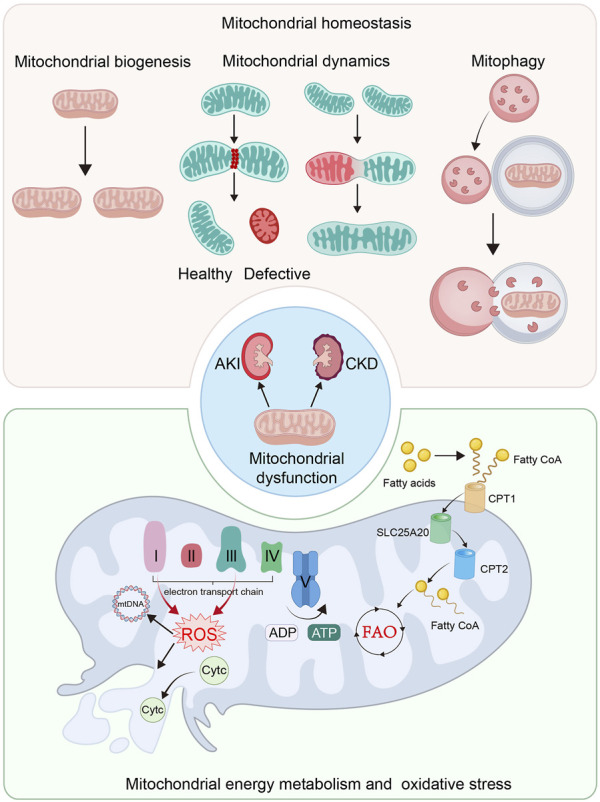
Mitochondrial dysfunction plays an important role in the pathological process of AKI and CKD. The imbalance of mitochondrial homeostasis, defective mitochondrial energy metabolism and mitochondrial oxidative stress are the crucial causes of mitochondrial dysfunction. The imbalance of mitochondrial homeostasis includes the impaired mitochondrial biogenesis, imbalance of mitochondrial dynamics and insufficient mitophgy. Mitochondria is the main organelle for energy production. Defective mitochondrial energy metabolism, including FAO and OXPHOS, plays a significant role in mitochondrial dysfunction. Mitochondrial energy metabolism not only induces ATP production insufficiency, but also in ROS production and mitochondrial impairment.

Mitochondrial quality control (MQC) is an elaborate mechanism to maintain homeostasis, ensuring the enough numbers of functional mitochondria (Pickles et al., 2018; Huang et al., 2022b). Involving mitochondrial biogenesis, dynamics, and mitophagy, mitochondrial homeostasis is intimately correlated with normal kidney function (Rahman et al., 2022) (Figure 1). Large reports have shown mitochondrial dysfunction is closely related to the progression of kidney diseases (Galvan et al., 2017). In both AKI and CKD models, mitochondrial biogenesis is greatly impaired, leading to the insufficient supply of ATP and cell injury in various cell types, particularly in TECs in kidney, (Galvan et al., 2017; Zhang et al., 2021a).

Mitochondrial structure and morphology plays a key role in energy production. They are tightly controlled by mitochondrial dynamics and mitophagy (Rahman et al., 2022). With a highly dynamic action, mitochondria frequently remodel themselves through fusion and fission cycles (Forbes and Thorburn, 2018; Mu et al., 2020; Liang et al., 2021). Mitochondrial fusion enables defective mitochondria mixing with healthy ones to repair (Forbes and Thorburn, 2018; Mu et al., 2020; Liang et al., 2021), while fission can remove their defective contents to keep healthy mitochondrial function (Aparicio-Trejo et al., 2018; Forbes and Thorburn, 2018; Mu et al., 2020; Liang et al., 2021). Furthermore, after losing membrane potentials, defective mitochondria would be degraded by mitophagy (Forbes and Thorburn, 2018; Jin et al., 2021). When this balance is disrupted, mitochondrial dysfunction occurs, which plays a significant role in the progression of kidney diseases (Galvan et al., 2017; Zhang et al., 2021a).

### 2.2 Defective energy metabolism and increased oxidative stress in mitochondria

To execute energy production and substance metabolism are the main functions of mitochondria (Spinelli and Haigis, 2018; Liang et al., 2021). The production of ATP mainly depends on aerobic respiration (Bhargava and Schnellmann, 2017). The pyruvate is derived from the process of glycolysis in cytoplasm, and then is converted into acetyl-coenzyme A (acetyl-CoA) to participate tricarboxylic acid (TCA) cycle in mitochondrial matrix. Furthermore, TCA cycle releases NAD^+^ to ultimately initiate OXPHOS. The OXPHOS system comprises complex I to V. Electrons are delivered by complex I and II in inner mitochondrial membrane (IMM), and then transmit to complex IV and are accepted by oxygen. When electrons travel through complex I, III, and IV, protons are actively flowing into intermembrane space. Complex V, the ATP synthase, generates ATP from ADP through the electrochemical gradient of protons. When this process is damaged, some electrons would leak and lead to ROS production (Bhargava and Schnellmann, 2017; Mani et al., 2020; Liang et al., 2021). Hence, mitochondria are also the main source of cellular ROS (Sinha et al., 2013). At physiological concentrations, ROS is important to cellular division, but excessive ROS leads to stressed damages to protein, DNA and lipid (Mani et al., 2020). Of note, mitochondria not only are the main generator of ROS, but always are the primary targets of ROS. ROS could impair mitochondrial DNA, ETC., and damage mitochondrial membrane, further leading to more production of ROS (Szczepanowska et al., 2012; Mani et al., 2020). Excessive ROS could lead to the depolarization of IMM, the opening of mitochondrial permeability transition pore (mPTP), and the leakage of cytochrome c (Cytc) into the cytoplasm. Furthermore, caspase signaling pathway would be activated to induce cell apoptosis and the development of kidney diseases (Sinha et al., 2013; Galvan et al., 2017; Mani et al., 2020; Zhang et al., 2021a; Rahman et al., 2022).

As a major cell type in kidney, proximal tubular cell produce ATP *via* fatty acid β-oxidation (FAO) in mitochondria (Bhargava and Schnellmann, 2017). The fatty acids are transferred from cytoplasm into mitochondrial matrix by carnitine O-palmitoyltransferase 1 (CPT1), carnitine–acylcarnitine translocase (SLC25A20) and carnitine O-palmitoyltransferase 2 (CPT2) to be metabolized for energy production (Houten and Wanders, 2010; Bhargava and Schnellmann, 2017; Spinelli and Haigis, 2018). Peroxisome proliferator–activated receptor α (PPARα), a crucial transcriptional factor, regulates FAO *via* controlling the transcription and translation of FAO-related enzymes (Chung et al., 2018; Barone et al., 2019). The impaired mitochondrial FAO leads to insufficient supply of ATP, but also results in lipotoxicity, which together contribute to renal fibrosis (Lv et al., 2019; Miguel et al., 2021). In both AKI and CKD models, the FAO failure and the following lipid accumulation commonly occur in renal TECs (Rong et al., 2021; Xu et al., 2022), which could further trigger the impairment of mitochondrial structure and function (Huang et al., 2022b).

## 3 Natural products for regulating mitochondrial function

### 3.1 Enhancing mitochondrial biogenesis and energetics

**FIGURE 2:**
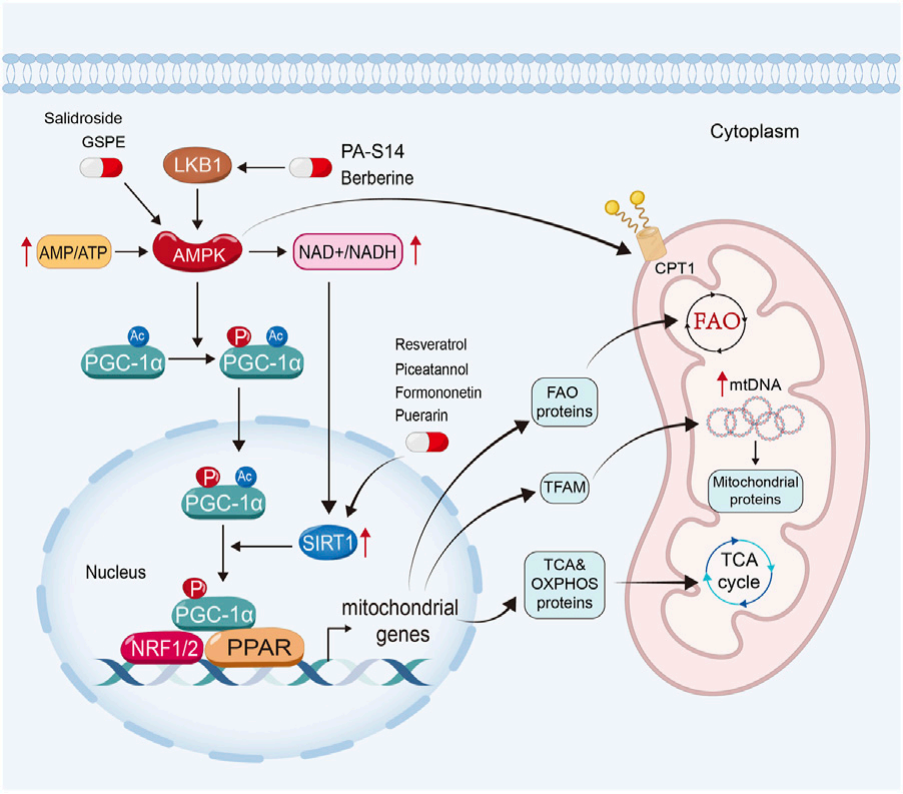
Enhancing mitochondrial biogenesis and energetics is a crucial therapeutic strategy for natural products to restore mitochondrial function in kidney diseases. PA-S14 and berberine promote mitochondrial biogenesis and energetics by activating LKB1, while salidroside and GSPE induce AMPK. Moreover, resveratrol, piceatannol, formononetin and puerarin exert their mitochondrial protective function *via* the activation of Sirtuin-1.

It is widely found that impaired mitochondrial biogenesis and energetics contribute to the pathogenesis of kidney diseases (Weinberg, 2011; Bhargava and Schnellmann, 2017). Mitochondrial biogenesis involves the growth and binary fission of pre-existing mitochondria (Jornayvaz and Shulman, 2010) to supply functional mitochondria for cellular requirement (Figure 2).

AMP-activated protein kinase (AMPK), Sirtuin-1, and peroxisome proliferator-activated receptor-γ (PPARγ) coactivator-1α (PGC-1α) in particular, play important roles in mitochondrial biogenesis (Jornayvaz and Shulman, 2010; Liang et al., 2021). In eukaryotic cells, when there is a deficiency of functional mitochondria, the AMP/ATP ratio increases to activate AMPK to induce the expression and phosphorylated activation of PGC-1α. Moreover, AMPK can enhance Sirtuin-1, the NAD^+^-dependent deacetylases family member owning deacetylating activity (Wang et al., 2019), through increasing cellular nicotinamide adenine dinucleotide (NAD^+^) levels. Sirtuin-1 is the main deacetylase of PGC-1α (Rodgers et al., 2005; Gerhart-Hines et al., 2007). After deacetylation and phosphorylation, PGC-1α translocates from the cytoplasm into the nucleus. As a co-activator, PGC-1α binds with several transcription factors (Galvan et al., 2017) and activates them. As reported, PGC-1α could activate nuclear respiratory factors (NRF) 1 and NRF2, estrogen-related receptors (ERRs), and PPARs (Jornayvaz and Shulman, 2010).

PGC-1α could dock on NRF 1 and 2 to together drive the transcription and translation of the genes involving TCA cycle and OXPHOS systems, and then induce mitochondrial biogenesis. Furthermore, it also induces the expression of mitochondrial transcription factor A (TFAM), a controller in replication and transcription of mitochondrial DNA, to further drive mitochondrial biogenesis (Jornayvaz and Shulman, 2010; Liang et al., 2021). PGC-1α also co-activates ERRs to enhance mitochondrial biogenesis and glucose utilization (Jornayvaz and Shulman, 2010; Deblois et al., 2013; Singh et al., 2018). Moreover, PGC-1α also interacts with PPARα, the master regulator of FAO to upregulate the expression of FAO-related genes, such as CPT1, SLC25A20 and CPT2 (Djouadi et al., 2005; Tachibana et al., 2009; Jornayvaz and Shulman, 2010; Galvan et al., 2017; Suzuki et al., 2021). Furthermore, AMPK can also promote mitochondrial FAO to enhance the activity of CPT1 by PGC-1α-independent pathway (Herzig and Shaw, 2018).

#### 3.1.1 AMPK signaling pathway activators

##### 3.1.1.1 Piericidin analogue S14 (PA-S14)

Many natural products can stimulate mitochondrial biogenesis and energetics *via* AMPK signaling pathway. PA-S14, a natural product extracted from the marine-derived *Streptomyces* reported by our group, is an analogue of Piericidin A, a mitochondrial complex I inhibitor. PA-S14 can activate liver kinase B1 (LKB1), the key upstream kinase for AMPK activation, with no inhibitory effects on mitochondrial complex I. We found that PA-S14 promotes mitochondrial biogenesis *via* LKB1-AMPK-PGC-1α signaling pathway, which protects against renal TECs cell senescence, renal fibrosis in various CKD models, such as unilateral ureteral obstruction (UUO), unilateral ischemia-reperfusion injury (UIRI), adriamycin nephropathy and 5/6 nephrectomized model (Liu et al., 2022a) (Table 1).

**TABLE 1:** Therapeutic mechanisms and targets of natural products targeting mitochondria in kidney diseases.

Targets		Natural products	Cell types	Animal model	References
Enhancing mitochondrial biogenesis and energetics	AMPK signaling pathway activators	PA-S14	TECs	UIRI, UUO, adriamycin nephropathy and 5/6 nephrectomized model	Liu et al. (2022a)
		Berberine	TECs, podocytes	DKD	Qin et al. (2020), Rong et al. (2021)
		Salidroside	Podocytes, mesangial cells	DKD	Xue et al., 2019; Shati (2020), Li et al. (2021b)
		GSPE	podocytes	Streptozotocin-and high-carbohydrate/high-fat diet-induced diabetic rats	Bao et al. (2014)
	Sirtuin activators	Resveratrol	TECs, mesangial cells and podocytes	Aging-related progressive renal injury, AKI and CKD	Wang et al. (2015b), Fu et al. (2017), Hui et al. (2017), Kim et al. (2018), Zhang et al. (2019), Zhang et al. (2020)
		Piceatannol	TECs	gamma-radiation-induced kidney injury	Mahmoud Moustafa et al. (2021)
		Formononetin	TECs, podocytes	DKD	Huang et al. (2022e)
		Puerarin	TECs	DKD, cadminum-induced kidney injury	Xu et al. (2016b), Song et al. (2016)
Alleviating mitochondrial oxidative stress	Keap1-Nrf2-ARE signaling pathway activators	EGCG	TECs	Cisplatin-induced kidney injury	Sahin et al. (2010), Pan et al. (2015)
		Capsaicin	TECs	Cisplatin-induced kidney injury	Kursunluoglu et al. (2014), Ran et al. (2022)
		Sulforaphane	TECs	Age-related kidney injury and maleic acid-induced nephrophathy	Briones-Herrera et al. (2018), Mohammad et al. (2022)
		Thymoquinone	TECs	AKI	Hannan et al. (2021), Hashem et al. (2021)
		Hesperetin	TECs	AKI	Wang et al. (2013), Kumar et al. (2017), Chen et al. (2019a)
		Astragaloside IV	TECs	Tacrolimus-induced chronic nephrotoxicity, cisplatin-and sepsis-induced AKI, IRI	Yan et al. (2017), Gao et al. (2020a), Feng et al. (2022), Su et al. (2022)
	Sirtuin activators	Curcumin	TECs	Aristolochic acid nephropathy and cisplatin-induced AKI	Ugur et al. (2015), Ortega-Domínguez et al. (2017), Liu et al. (2022c)
		Resveratrol	TECs and mesangial cells	Aging-related progressive renal injury, cadminum-and sepsis-induced kidney injury, and DKD	Xu et al. (2012), Wang et al. (2015b), Fu et al. (2017), Kim et al. (2018), Wang et al. (2018), Rahman et al. (2022)
	Antioxidant	L-Carnitine	TECs	UUO and DKD	Ye et al. (2010), Zhao et al. (2021a), Ito et al. (2022)
		CAPE	TECs	IRI and cadmium-induced AKI	Kobroob et al. (2012), Ashkar et al. (2022)
		Astaxanthin	Mesangial cells	DKD and bisphenol A-induced kidney toxicity	Jiang et al. (2020b), Chen et al. (2020), Naito et al. (2021)
Promoting mitophagy	Sirtuin activators	Polydatin	TECs	Sepsis-induced AKI	Gao et al. (2020b)
		Quercetin	TECs	UUO	Liu et al. (2020)
	Nrf2 activators	Urolithin A	TECs	Fructose-fed mice	Zhang et al. (2022a)
	Receptors mediated mitophagy activators	PNS	TECs	Cisplatin induced AKI	Liang et al. (2017), Li et al. (2021c)
Regulating mitochondrial dynamics	Sirtuin activators	Almost natural sirtuin activators mentioned above			
	Nrf2 activators	Hyperoside	TECs and podocytes	Adriamycin nephropathy and IRI	Chen et al. (2017), Wu et al. (2019)

##### 3.1.1.2 Berberine

Berberine, found in *Coptidis rhizoma* and *Cortex Phellodendri*, can activate AMPK *via* LKB1-dependent or independent pathway (Joshi et al., 2019). Berberine can alleviate mitochondrial dysfunction in DKD mice and palmitic acid-induced podocyte injury *via* increasing AMPK and PGC-1α (Qin et al., 2020; Li et al., 2021b). Moreover, berberine can also improve FAO and rescue mitochondrial function by inducing CPT1 and CPT2 in podocytes, which further attenuates lipid accumulation and ROS overproduction in DKD (Qin et al., 2020). Berberine treatment also decreases the excretion of urinary microalbumin (Qin et al., 2020). Besides podocytes, berberine can also upregulate the expression of CPT1 and PPARα in renal TECs, further improving mitochondrial FAO and reducing lipid accumulation in DKD mice (Rong et al., 2021). In one clinical trial, berberine decreases albumin-to-creatine ratio and improves renal hemodynamics in hypertensive patients with type 2 diabetes mellitus (Dai et al., 2015).

##### 3.1.1.3 Salidroside

Salidroside, a phenolic glucoside found in *Rhodiola rosea* (Liang et al., 2021), exhibits versatile pharmacologic properties, such as anti-apoptosis, anti-inflammation, and improving mitochondrial function (Wang et al., 2022). Salidroside also shows great protective effects on kidney function maintaining (Huang et al., 2019; Fan et al., 2022). The underlying mechanism lies on its activity to stimulate AMPK-Sirtuin-1-PGC-1α signaling (Xue et al., 2019; Shati, 2020; Li et al., 2021b) and promotes mitochondrial biogenesis (Xue et al., 2019).

##### 3.1.1.4 Grape seed proanthocyanidin extract (GSPE)

GSPE, a natural bioactive compound from plant, exhibits great therapeutic properties through inhibiting oxidative stress and promoting mitochondrial biogenesis (Ashkar et al., 2022). Researches show in streptozotocin-and high-carbohydrate/high-fat diet-induced diabetic rats, GSPE treatment is able to enhance mitochondrial biogenesis, and ameliorate podocyte injury and apoptosis *via* AMPK-Sirtuin-1-PGC-1α-TFAM signaling pathway (Bao et al., 2014).

#### 3.1.2 Sirtuin activators

##### 3.1.2.1 Resveratrol

Resveratrol, a natural compound extracted from the berries of *Vaccinium* species, can activate sirtuin-1, sirtuin-3, and sirtuin-5 (Dai et al., 2018). Clinical studies have shown that resveratrol exhibits therapeutic effects on several diseases, including cardiovascular disease and neurological diseases (Xia et al., 2017; Singh et al., 2019). In kidney diseases, resveratrol could safely improve peritoneal ultrafiltration activity and ameliorate renal dysfunction (Lin et al., 2016; Singh et al., 2019). The mechanisms are because resveratrol can activate Sirtuin-1 to further promote activation of PGC-1α and mitochondrial biogenesis. Resveratrol can also regulate mitochondrial biogenesis *via* Sirtuin-1-independent pathway, such as resveratrol can directly activate AMPK (Ungvari et al., 2011; Joshi et al., 2019). The protective effects of resveratrol are shown in various models of kidney diseases, including aging-related progressive renal injury, AKI and CKD (Wang et al., 2015b; Fu et al., 2017; Hui et al., 2017; Kim et al., 2018; Zhang et al., 2019; Zhang et al., 2020). To induce Sirtuin-1-PGC-1α-PPARα pathway, resveratrol can also enhance mitochondrial FAO function in renal cells (Kim et al., 2013).

##### 3.1.2.2 Piceatannol

Piceatannol, a natural phytochemical from various fruits and vegetables, is an activator of sirtuin-1. Piceatannol exhibits versatile pharmacologic therapeutic potencies in cancer, cardiovascular diseases and atherosclerosis, as well as in kidney diseases (Tovar-Palacio et al., 2022). Piceatannol can ameliorate necrosis and apoptosis in renal TECs through activating Sirtuin-1-PGC-1α pathway (Mahmoud Moustafa et al., 2021).

##### 3.1.2.3 Formononetin

Formononetin, the major phytochemical of the *Astragalus membranaceus* (Tovar-Palacio et al., 2022), can also upregulate and activate Sirtuin-1 (Rasbach and Schnellmann, 2008). Formononetin is famous for its therapeutic effects on cardiac diseases and hyperlipidemia (Tovar-Palacio et al., 2022). Reports have also demonstrated its protective effects in kidney. Formononetin can alleviate nephrotoxicity in methotrexate-, cisplatin-and gentamicin-induced kidney injury and DKD (Aladaileh et al., 2019; Oza and Kulkarni, 2019; Hao et al., 2021; Althunibat et al., 2022). Formononetin promotes mitochondrial biogenesis *via* Sirtuin-1-PGC-1α pathway, which plays an important role in kidney protection (Rasbach and Schnellmann, 2008; Huang et al., 2022e). Formononetin treatment could attenuate tubular cell apoptosis and podocyte injury, then further to decrease urinary albumin excretion and ameliorate the progression of DKD (Huang et al., 2022e).

##### 3.1.2.4 Puerarin

Puerarin, the bioactive compound extracted from *radix puerariae*, exhibits beneficial therapeutic effects on cardiovascular diseases, cancer, diabetes and osteonecrosis. Puerarin has wide pharmacological properties, especially the activation of Sirtuin-1 (Zhou et al., 2014; Tovar-Palacio et al., 2022). Puerarin can ameliorate mitochondrial damage to inhibit cadminum-induced TEC apoptosis in kidney (Song et al., 2016). Moreover, puerarin upregulates Sirtuin-1 and PGC-1α expression in DKD, to further ameliorate matrix expansion, glomerular collapse and tubular dilatation, and rescue kidney function (Xu et al., 2016b). Puerarin can also activate LKB1 and AMPK to further promote mitochondrial biogenesis in kidney (Song et al., 2017; Li et al., 2020). In clinical trials, puerarin could also significantly decrease the urinary albumin excretion rate in patients with DKD (Wang et al., 2015a; Shao et al., 2021).

### 3.2 Alleviating mitochondrial oxidative stress

**FIGURE 3:**
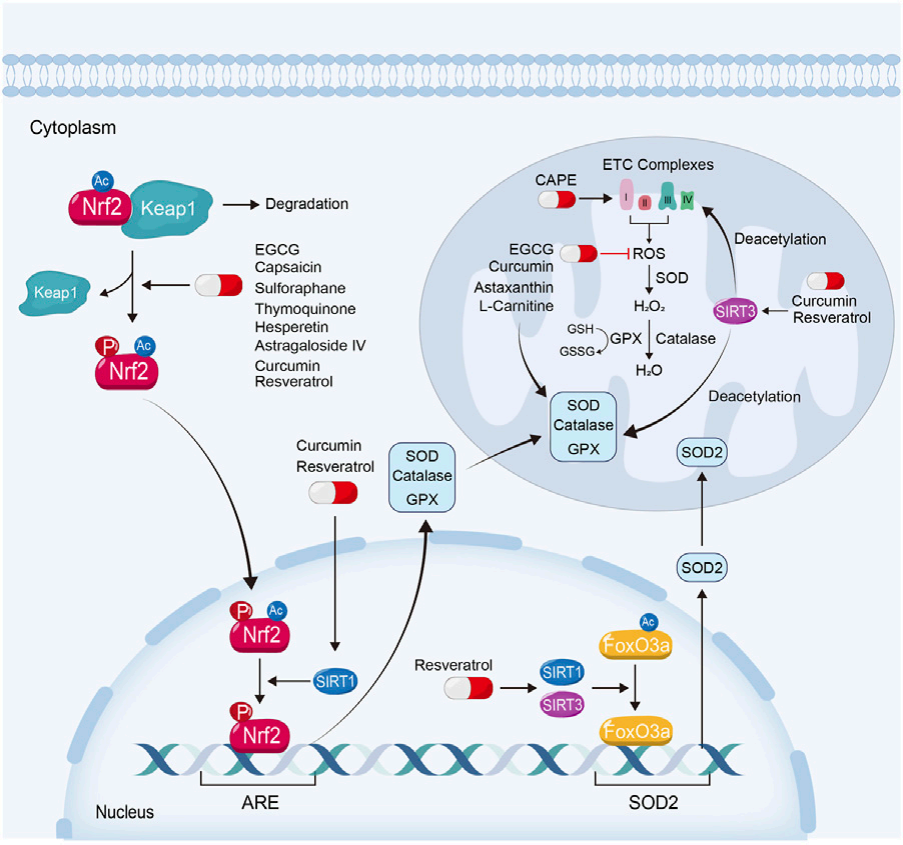
Attenuating mitochondrial oxidative stress is also an important way for natural products to ameliorate kidney injury. EGCG, capsaicin, sulforaphane, thymoquinone, hesperetin and astragaloside IV, exhibit their renoprotective effects *via* the activation of Keap1-Nrf2-ARE signaling pathway. Curcumin and resveratrol ameliorate mitochondrial dysfunction in kidney diseases not only depending on Keap1-Nrf2-ARE signaling pathway, but also on the activation of Sirtuin-1 and Sirtuin-3. Moreover, some natural products protect against mitochondrial oxidative stress by scavenging ROS, reducing mitochondrial ROS production or restoring anti-oxidative enzyme, such as L-Carnitine, CAPE, EGCG, curcumin and astaxanthin.

Mitochondrial respiration is the main source of cellular ATP. The impairment and uncoupling of ETC leads to the excessive generation of ROS, which further trigger a series of cellular injuries. As the primary source of cellular ROS (Sinha et al., 2013), mitochondria also own the adequate anti-oxidant system. Superoxide dismutase (SOD) plays a major role in eliminating mitochondrial ROS. SOD could convert ROS into hydrogen dioxide, which is then deactivated by catalase or glutathione (GSH). GSH could also be converted to oxidized GSH (GSSG) *via* various glutathione peroxidases (GPX) in this process (Balaban et al., 2005) (Figure 3).

Nuclear factor-erythroid-derived 2-like 2 (NFE2L2, also named Nrf2) is a transcription factor which plays a crucial role in resistance to oxidative stress (Ma, 2013). Under basal conditions, Nrf2 is degraded through kelch-like erythroid cell-derived protein with CNC homology-associated protein 1 (Keap1)-dependent ubiquitination and proteasomal degradation. When the binding of Keap1 and Nrf2 is disrupted, Nrf2 translocates into the nucleus, where it binds with a common DNA sequence of antioxidant response element (ARE). ARE involves various genes of anti-oxidative stress function, such as SOD, GPX and catalase (Ma, 2013; Dinkova-Kostova and Abramov, 2015; Ryoo and Kwak, 2018; Wang et al., 2020a). Nrf2 can be phosphorylated by AMPK and deacetylated by Sirtuin-1, and reversely enhanced the transcriptional activity by them (Gureev et al., 2019; Xu et al., 2021). Of note, to reduce the generation of mitochondrial ROS or to increase the scavenge of ROS similarly attenuate mitochondrial oxidative stress and kidney injury (Li et al., 2021b).

#### 3.2.1 Keap1-Nrf2-ARE signaling pathway activators

##### 3.2.1.1 Epigallocatechin 3-Gallate (EGCG)

EGCG, a bioactive constituent extracted from green tea, is widely investigated in various diseases, such as cardiovascular diseases, diabetes and cancer. EGCG exhibits anti-oxidation properties and therapeutic effects against AKI, CKD, kidney stone diseases, glomerulonephritis, lupus nephritis, and renal cell carcinoma (Kanlaya and Thongboonkerd, 2019; Li et al., 2021b). In one clinical trial, EGCG is able to decrease albuminuria and ameliorate DKD (Avila-Carrasco et al., 2021). Studies have shown that EGCG inhibits mitochondrial oxidative stress and ameliorates kidney injury *via* inhibiting Keap1 and upregulating Nrf2-ARE signaling pathway (Wang et al., 2015c; Granata et al., 2015; Pan et al., 2015; Kanlaya et al., 2016; Sun et al., 2017). There are two mechanisms for EGCG in enhancing mitochondrial antioxidant defense. First, EGCG can reduce mitochondrial ROS, then to further prevent mitochondrial respiratory enzymes injury. Second, EGCG could upregulate Keap1-Nrf2-ARE pathway, to further induce the expression of SOD, GPX, GSH and catalase. Hence, EGCG protects mitochondrial respiratory enzymes and decreases mitochondrial ROS production in cisplatin-induced nephrotoxicity (Sahin et al., 2010; Pan et al., 2015). EGCG treatment also significantly reverses the increased levels of Scr and BUN, and alleviates tubular injury in cisplatin-induced kidney injury (Pan et al., 2015).

##### 3.2.1.2 Capsaicin

Capsaicin, the primary bioactive compound from red chili peppers (Liu et al., 2022d), exhibits therapeutic effects on kidney in various animal models, such as UUO (Liu et al., 2022d), DKD (Ríos-Silva et al., 2018) and nephrotoxicity (Jung et al., 2014). Capsaicin can activate Nrf2-ARE signaling pathway and rescue kidney function in cisplatin-induced kidney injury. Capsaicin treatment enhances the activities of mitochondrial ROS scavenging enzymes and ameliorates mitochondrial swelling and fragmentation, and prevents TECs apoptosis (Kursunluoglu et al., 2014; Ran et al., 2022). In clinical trials, capsaicin also shows to alleviate uremic conditions in patients with CKD (Makhlough et al., 2010; Yeam et al., 2021).

##### 3.2.1.3 Sulforaphane

Sulforaphane, extracted from *Brassicae* vegetables, can activate Nrf2 signaling pathway and protect against mitochondrial oxidative stress (Briones-Herrera et al., 2018). Sulforaphane shows great renoprotective effects *via* targeting mitochondrial dysfunction in various kidney diseases. A previous study showed that sulforaphane treatment could increase SOD to decrease mitochondrial damage and preserve mitochondrial microstructure after kidney transplantation (Cekauskas et al., 2013). Sulforaphane can also alleviate age-related mitochondrial dysfunction and kidney injury *via* inhibiting Keap1 and upregulating Nrf2 (Mohammad et al., 2022). In maleic acid-induced nephropathy, sulforaphane rescues mitochondrial function and alleviates kidney injury. Sulforaphane could also improve mitochondrial electron transport system to alleviate mitochondrial oxidative stress through promoting Nrf2-induced ROS scavenging (Briones-Herrera et al., 2018).

##### 3.2.1.4 Thymoquinone

Thymoquinone, the main bioactive compound from *Nigella sativa* seed and its oil, exhibits protective effects in various organs. Clinical trials have shown that *N. sativa* oil can reduce Scr levels and delay the progression of CKD. Moreover, it can also decrease the size of kidney stones (Hannan et al., 2021). Thymoquinone protects against drug-induced kidney injury, ischemia-reperfusion injury (IRI) and CKD *via* its anti-oxidative, anti-inflammatory and anti-fibrotic properties (Hannan et al., 2021). Thymoquinone can increase the expression of mitochondrial ROS scavenging enzymes and antioxidant molecules, such as GPX, catalase, SOD and GSH, and restore renal mitochondrial viability *via* Nrf2 in various kidney diseases models (Hannan et al., 2021; Hashem et al., 2021). Thymoquinone is regarded as a mitochondria-targeted antioxidant (Granata et al., 2015). Hilal Alkis et al. found that thymoquinone can alleviate radiation-induced oxidative stress in kidney tissue in rats (Alkis et al., 2021). Another study has shown that thymoquinone attenuates doxorubicin-induced redox imbalance in renal tissue *via* the upregulation and activation of Nrf2 (Elsherbiny and El-Sherbiny, 2014). Other studies also found that thymoquinone upregulates mitochondrial anti-oxidative ability, rescues mitochondrial function and ameliorates kidney injury through Nrf2 (Dera et al., 2020; Hashem et al., 2021).

##### 3.2.1.5 Hesperetin

Hesperetin, a natural bioactive phytochemical from citrus plants, exhibits wide pharmacological therapeutic effects on cancer, cardiovascular diseases and diabetes (Chen et al., 2019b; Ashkar et al., 2022). Hesperetin also shows renal protective effects by its anti-oxidative properties (Ashkar et al., 2022). Hesperetin can activate Nrf2-ARE signaling pathway *via* increasing Nrf2 and p-Nrf2 levels in kidney tissue (Chen et al., 2019b). Hesperetin treatment can also significantly rescue kidney function and inhibit tubular cell apoptosis in various AKI models, through increasing mitochondrial ROS scavenging enzymes *via* Nrf2-ARE signaling pathway (Wang et al., 2013; Kumar et al., 2017; Chen et al., 2019a).

##### 3.2.1.6 Astragaloside IV

*A. membranaceus*, is a traditional Chinese herbs used in diverse kidney diseases. Astragaloside IV, one of the main bioactive ingredients isolated from *A. membranaceus*, exhibits significant protective effects in kidney (Gao et al., 2020a). Mitochondrial oxidative defense is one of the main mechanisms of astragaloside IV to restore kidney function. It lies in its effects on increasing Nrf2 protein levels and promoting Nrf2 transcriptional activity (Gao et al., 2020a). Studies have found that astragaloside IV can also induce the expression of GPX, SOD, as well as catalase, and ameliorate mitochondrial oxidative stress *via* Keap1-Nrf2-ARE signaling pathway in AKI and CKD models (Yan et al., 2017; Gao et al., 2020a; Feng et al., 2022; Su et al., 2022).

#### 3.2.2 Sirtuin activators

Sirtuin activators not only play important roles in mitochondrial biogenesis, but also in mitochondrial oxidation defense. Sirtuin-1 can promote Nrf2 deacetylation and enhance its transcriptional activity, further protecting against mitochondrial oxidative stress (Xu et al., 2021). Sirtuin-3, a nuclear protein translocated to the mitochondrial matrix upon oxidative stress (Klotz et al., 2015), plays an important role in mitochondrial bioenergetics and defense to oxidative stress. On one hand, Sirtuin-3 is able to deacetylate the complexes of the ETC and increase their activities to further reduce the production of ROS. On the other hand, Sirtuin-3 can deacetylate mitochondrial proteins involved in the elimination of ROS to reduce mitochondrial ROS levels (Clark and Parikh, 2021). Moreover, both Sirtuin-3 and Sirtuin-1 can deacetylate and activate forkhead box class O 3a (FoxO3a), the ubiquitously expressed transcription factors, and then upregulating the genes involved in mitochondrial oxidative defense, such as SOD2 (Jacobs et al., 2008; Tseng et al., 2013; Klotz et al., 2015; Liu et al., 2018; Xie et al., 2020; Li et al., 2021a; Ni et al., 2021; Rahman et al., 2022). Sirtuin-3 can also rescue kidney function by relieving mitochondrial oxidative stress (Fu et al., 2017).

##### 3.2.2.1 Curcumin

Curcumin, the bioactive compound extracted from *turmeric*, shows the potent therapeutic effects in various diseases including renal IRI, shock and myocardial ischemia with its antioxidant properties (Trujillo et al., 2013). Curcumin is a powerful bifunctional antioxidant. On one hand, it exhibits renoprotective effects and improves mitochondrial function through scavenging mitochondrial ROS directly (Trujillo et al., 2013). On the other hand, it can alleviate mitochondrial oxidative stress *via* the activation of Nrf2, Sirtuin-1 and Sirtuin-3 (Molina-Jijón et al., 2011; Tapia et al., 2012; Martínez-Morúa et al., 2013; Trujillo et al., 2013; Huang et al., 2022a; Liu et al., 2022c). Curcumin can activate Nrf2 signaling pathway *via* inhibiting Keap1, upregulating Nrf2 and inducing the nuclear translocation of Nrf2 (Ashrafizadeh et al., 2020). Moreover, a study also has shown that curcumin alleviates aristolochic acid-induced nephropathy and tubular cell apoptosis *via* Sirtuin-1-Nrf2-ARE signaling pathway (Liu et al., 2022c). Curcumin can also upregulate the expression of SOD, GPX and catalase *via* Nrf2 to ameliorate mitochondrial oxidative stress, and further restore the activities of renal mitochondrial respiratory enzyme in various kidney diseases (Trujillo et al., 2013; Liu et al., 2022c). Curcumin also induces Sirtuin-3 for renoprotective effects in cisplatin-induced kidney injury (Ugur et al., 2015; Ortega-Domínguez et al., 2017). Clinical trials showed that curcumin can attenuate proteinuria in patients with DKD and lupus nephritis (Khajehdehi et al., 2011; Khajehdehi et al., 2012). In patients with end stage renal disease, curcumin can also alleviate uremic condition (Pakfetrat et al., 2014).

##### 3.2.2.2 Resveratrol

Besides promoting mitochondrial biogenesis, resveratrol also attenuates mitochondrial dysfunction and restores renal function *via* other multiple mechanisms. Resveratrol is also a natural product with anti-oxidative properties. Resveratrol can promote the nuclear translocation of Nrf2 by inhibiting Keap1 expression (Farkhondeh et al., 2020). Moreover, resveratrol activates sirtuin-1 to activate Nrf2 pathway and protect against oxidative stress (Zhang et al., 2022b). Furthermore, it exhibits anti-oxidation properties *via* the activation of Sirtuin-3. On one hand, Sirtuin-3 can regulate SOD2 expression *via* Sirtuin-3-Foxo3a-SOD2 pathway. On the other hand, Sirtuin-3 can also deacetylate and activate mitochondrial proteins involved in the elimination of ROS (Xu et al., 2016a; Clark and Parikh, 2021). Studies have found that resveratrol can upregulate the expression of mitochondrial ROS scavenging enzymes, and attenuate mitochondrial oxidative stress *via* the activation of Nrf2 pathway and Sirtuin-3-FoxO3a pathway in AKI and CKD models (Xu et al., 2012; Wang et al., 2015b; Fu et al., 2017; Kim et al., 2018; Wang et al., 2018; Rahman et al., 2022).

#### 3.2.3 Antioxidant

##### 3.2.3.1 L-carnitine

L-carnitine mainly derives from daily diet (Granata et al., 2015). Abundant studies have demonstrated that L-carnitine exhibits protective effects for its antioxidant properties through targeting mitochondria (Zhao et al., 2021a). L-carnitine can improve mitochondrial respiratory chain activity and decrease mitochondrial ROS production *via* promoting acetyl-CoA production (Zhao et al., 2021a). Moreover, L-carnitine can also directly scavenge ROS and protect against the ETC complex impairment. Furthermore, L-carnitine can also restore anti-oxidative molecules and enzymes, such as GSH, SOD, catalase and GPX, in kidney diseases (Ye et al., 2010; Granata et al., 2015). Studies also showed L-carnitine treatment reduces mitochondrial ROS, ameliorates mitochondrial damage in renal TECs, alleviates renal tubulointerstitial fibrosis in UUO, and reduces urinary albumin excretion in DKD (Ye et al., 2010; Zhao et al., 2021a; Ito et al., 2022).

##### 3.2.3.2 Caffeic acid phenethyl ester (CAPE)

CAPE, a natural antioxidant, possesses beneficial roles in cadioprotective, neuroprotective and renoprotective effects (Kamarauskaite et al., 2021). Mitochondrial respiratory chain complex I and III are considered to be the main producers of ROS, the impairment of which contribute to mitochondrial ROS formation (Ashkar et al., 2022). CAPE exhibits a strong antioxidant potential on scavenging mitochondrial ROS (Ashkar et al., 2022). First, CAPE pretreatment protects complex I activity and inhibits ROS generation in complex II in renal IRI model. It can protect against outer mitochondrial membrane (OMM) damage and the release of Cytc from mitochondria. CAPE pretreatment also protects against necrotic cell death and cell apoptosis after renal IRI (Trumbeckaite et al., 2017; Ashkar et al., 2022). Second, CAPE treatment can restore the activity of mitochondrial antioxidant enzymes and increase GSH after kidney injury (Kobroob et al., 2012; Ashkar et al., 2022).

##### 3.2.3.3 Astaxanthin

Astaxanthin, a natural non-toxic product in most of marine organisms, exhibits wide biologic properties, such as strong antioxidant, anti-apoptotic and anti-inflammatory activities, for its unique structure (Chen et al., 2020; Giannaccare et al., 2020). Accumulated studies have demonstrated that astaxanthin exerts protective effects against oxidative stress-associated mitochondrial dysfunction (Kim and Kim, 2018). Study has shown that the antioxidant ability of astaxanthin is 100 times stronger than GSH, an important mitochondrial anti-oxidative molecule (Chen et al., 2020). Astaxanthin can easily span through the cell membrane, and then exhibits its antioxidant activities by scavenging ROS and enhancing the activities of antioxidant enzymes (Kim and Kim, 2018). Studies have demonstrated that astaxanthin treatment can alleviate mitochondrial dysfunction and rescue kidney function *via* its antioxidant activities in several kidney diseases, such as DKD (Chen et al., 2020; Naito et al., 2021) and bisphenol A-induced kidney toxicity (Jiang et al., 2020b).

### 3.3 Promoting mitophagy

Mitophagy is a specialized form of autophagy. When damaged mitochondria is unable to maintain their membrane potential, it needs to be degraded through mitophagy (Forbes and Thorburn, 2018; Jin et al., 2021). The defection of mitophagy will lead to the accumulation of dysfunctional mitochondria, resulting in the leakage of Cytc into the cytoplasm to induce the activation of the caspase signaling pathway, and contribute to cell death (Sinha et al., 2013; Mani et al., 2020; Rahman et al., 2022).

**FIGURE 4:**
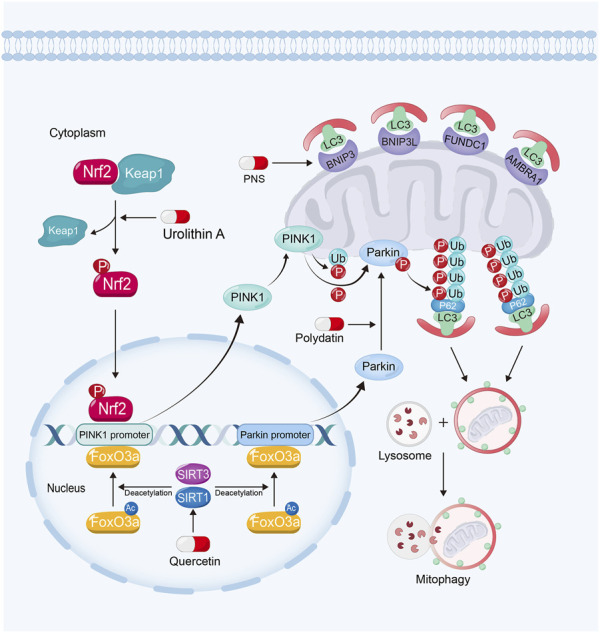
Polydatin promotes mitophagy by driving Parkin translocation from cytosol into OMM in kidney diseases. Quercetin can upregulate PINK1 and Parkin expression *via* sirtuin-1-FoxO3a-PINK1/Parkin signaling pathway. Urolithin A enhances mitophagy in kidney diseases by Nrf2-PINK1 pathway and PNS enhances mitophagy *via* the upregulation of BNIP3.

Mitophagy mainly consists of PTEN-induced kinase 1 (PINK1)/parkin RBR E3 ubiquitin-protein ligase (Parkin)-mediated and receptor-mediated mitophagy (Dagar et al., 2022). PINK1 and Parkin play important roles in the formation of autophagosome (Forbes and Thorburn, 2018). Under normal condition, PINK1 is transported from cytosol to mitochondrial matrix, where it is cleaved. When mitochondria lose their membrane potential, mitochondrial protein input is impaired, and PINK1 accumulates on OMM. And then it phosphorylates the pre-existing ubiquitin (Ub) on OMM, which recruits Parkin into OMM. The recruited Parkin on OMM is also phosphorylated by PINK1. Phosphorylated Parkin is able to ubiquitinate various proteins on OMM surface, which of them further are phosphorylated by PINK1. And then, Parkin is recruited and phosphorylated. It turns to be a feedforward cycle (Pickles et al., 2018; Jin et al., 2021). Then Ubs on OMM recruits ubiquitin-bond autophagy receptors like p62, which interacts with LC3 to form autophagosome (Kerr et al., 2017; Sun et al., 2021). These receptors, including B-cell lymphoma 2/adenovirus E1B 19-kDa-interacting protein 3 (BNIP3), BNIP3-like (BNIP3L), FUN14 domain-containing protein 1 (FUNDC1) and the activating molecule in BECN1-regulated autophagy protein 1 (AMBRA1), would bind with LC3 to form autophagosome (Dagar et al., 2022). Then the autophagosome containing injured mitochondria is transported to lysosome for degradation (Jin et al., 2021). It has been reported that enhanced mitophagy can relieve mitochondrial dysfunction and attenuate kidney injury (Xiao et al., 2017; Wang et al., 2020b; Zhang et al., 2021b) (Figure 4).

#### 3.3.1 Sirtuin activators

Sirtuins also play important roles in regulating mitophagy for their deacetylation activity (Zhao N. et al., 2021). Sirtuin-1 and Sirtuin-3 can deacetylate and activate FoxO3a. Furthermore, FoxO3a can bind to PINK1 and Parkin gene promoter and upregulate their expression to enhance mitophagy (Zheng et al., 2019; Zhao et al., 2021c; Ding et al., 2021; Guo et al., 2021; Liu et al., 2022b; Huang et al., 2022d; Gupta et al., 2022; Yao et al., 2022). Moreover, the activation of Sirtuin-1 can promote Parkin to translocate from cytosol into OMM, while inhibition of Sirtuin-1 reverses these effects (Gao et al., 2020b). However, the mechanism of this remains unknown.

##### 3.3.1.1 Polydatin

Polydatin, a resveratrol glycoside isolated from traditional Chinese herb, *Polygonum cuspidatum* (Gao et al., 2015), exhibits renoprotective effects *via* the activation of Sirtuin-1. For example, it has been found that polydatin promotes mitochondrial biogenesis and protects against mitochondrial oxidative stress *via* the activation of Sirtuin-1-PGC-1α pathway and Sirtuin-1-Nrf2-ARE pathway in kidney diseases (Huang et al., 2015; Zeng et al., 2016; Chen and Lan, 2017; Gao et al., 2020b). Polydatin can trigger the translocation of Parkin from cytosol into OMM, resulting in the enhanced mitophagy. Polydatin treatment alleviates tubular cell apoptosis and rescues renal function in sepsis-induced AKI (Gao et al., 2020b).

##### 3.3.1.2 Quercetin

Quercetin, a Sirtuin-1 and Sirtuin-3 activator, can be widely obtained from many fruits and vegetables, and exhibits wide biological effects in resisting inflammation, diabetic and microbial activities (Chen et al., 2021; Zymone et al., 2022). Quercetin also shows renoprotective effects against nephrotoxicity, AKI and CKD (Lu et al., 2021; Chen et al., 2022). It has been found quercetin can upregulate PINK1 and Parkin expression *via* Sirtuin-1 activation. Quercetin treatment also attenuates renal TEC senescence by promoting mitophagy (Liu et al., 2020).

#### 3.3.2 Nrf2 activators

The activation of Nrf2-ARE signaling pathway also plays an important role in protecting against mitochondrial dysfunction through promoting mitophagy (Ran et al., 2022). Nrf2 can bind to AREs on PINK1 promoter, and then upregulate PINK1 expression and promote mitophagy (Murata et al., 2015; Xiao et al., 2017; Gumeni et al., 2021; Chen, 2022).

##### 3.3.2.1 Urolithin A

Urolithin A, a natural gut metabolite of ellagic acid and ellagitannins, rich in nuts, is a safe mitophagy activator (Andreux et al., 2019; Jin et al., 2021). Accumulated evidences have shown that urolithin A exhibits multiple protective effects inducing mitophagy in heart (Huang et al., 2022c), muscle (Luan et al., 2021) and brain (Jayatunga et al., 2021). As a Nrf2 activator, urolithin A not only protects kidney against oxidative stress *via* promoting Nrf2 translocating into nucleus (Zhang et al., 2022c), but also ameliorates hyperuricemic nephropathy *via* enhancing PINK1-associated mitophagy in renal TECs (Zhang et al., 2022a). A study showed that urolithin A treatment restores renal function, and alleviates tubular cell dilation and collagen deposition in glomerular basement membrane (Zhang et al., 2022a).

#### 3.3.3 Receptors mediated mitophagy activators

Panax notoginseng saponins (PNS), the primary ingredient from Panax notoginseng, exhibits extensive therapeutic effects in cardiovascular diseases (Liu et al., 2014), lung cancer (Yang et al., 2016) and diabetes (Uzayisenga et al., 2014). Studies have demonstrated that PNS also exhibits powerful protective effects against DKD and AKI (Li et al., 2021c; He et al., 2022). It has been found that PNS is able to alleviate cisplatin-induced nephrotoxicity by inducing BNIP3-mediated mitophagy *via* hypoxia-inducible factor-1α (HIF-1α). PNS treatment restores renal function *via* attenuating the accumulation of injury mitochondria and cell apoptosis in TECs (Liang et al., 2017; Li et al., 2021c). In one clinical trial, PNS shows renoprotective effects on reducing albuminuria, proteinuria and Scr in patients with DKD (Tang et al., 2020).

### 3.4 Regulating mitochondrial dynamics

**FIGURE 5:**
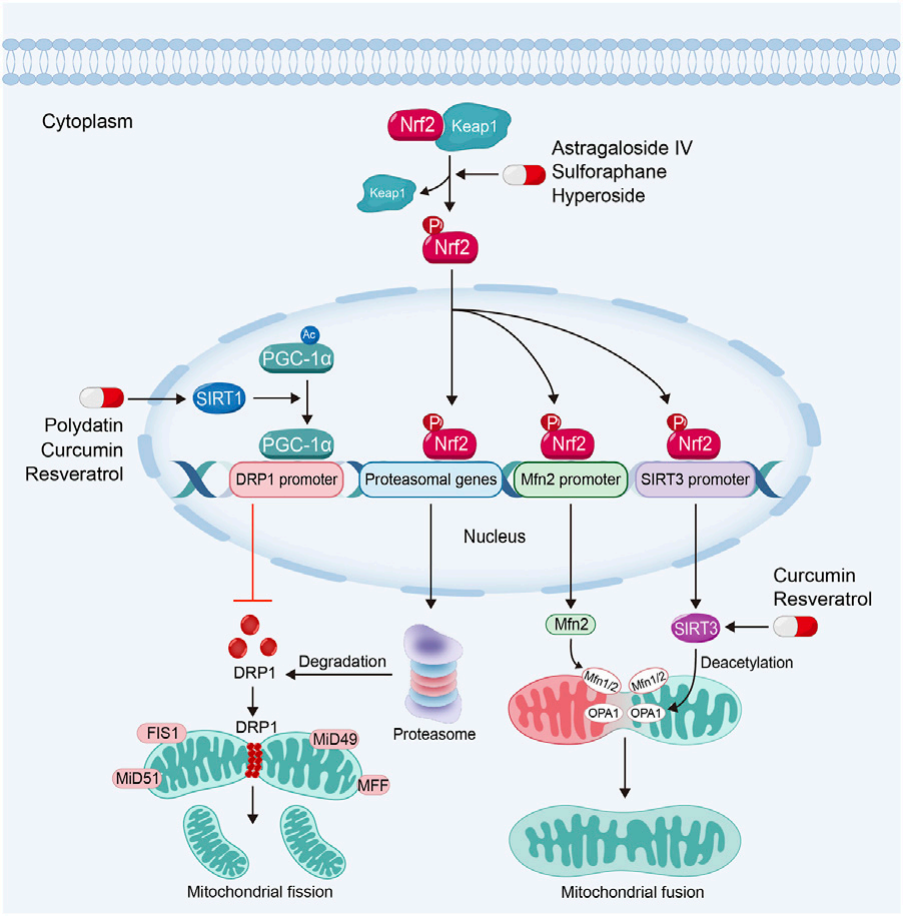
Sirtuin activators such as polydatin, resveratrol and curcumin regulate mitochondrial dynamics *via* sirtuin-1-PGC-1α-DRP1 pathway and the activation of OPA1 by sirtuin-3 in kidney diseases. Nrf2 activators inhibit mitochondrial fission and promote mitochondrial fusion in kidney diseases by Nrf2-proteasomal genes/Mfn2/sirtuin-3 signaling pathway.

As a dynamic organelle, mitochondria frequently change their shape to satisfy the variations in metabolic demands (Qin et al., 2019). The accumulation of dysfunctional mitochondria with abnormal morphology contributes to the progression of kidney diseases (Galvan et al., 2017). Mitochondrial dynamics plays important roles in maintaining mitochondrial structure and morphology (Rahman et al., 2022). The maintenance of mitochondrial dynamics involves the balance between mitochondrial fission and fusion. In kidney diseases, extensive mitochondrial fission leads to mitochondrial fragmentation and the insufficient function (Galvan et al., 2017) (Figure 5).

Dynamin related protein 1 (DRP1), the main mediator for mitochondrial fission, is recruited into OMM by its receptors, such as mitochondrial fission 1 protein (FIS1), mitochondrial fission factor (MFF) and mitochondrial dynamics proteins of 49 (MiD49) and 51. Then DRP1 constricts mitochondrial membrane and drives mitochondrial fission (Galvan et al., 2017). Upregulated DRP1 in kidney diseases can promote the permeability of OMM *via* the interaction with Bcl-2-associated X protein (BAX), a crucial executor of mitochondria-regulated apoptosis (Spitz and Gavathiotis, 2022) to trigger cell apoptosis (Qin et al., 2019). By contrast, the interaction of mitofusin 1 (Mfn1) and 2 mediates OMM fusion, while optic atrophy 1 (OPA1) regulates IMM fusion (Galvan et al., 2017). Studies have found that the downregulation of OPA1 will induce mitochondrial fragmentation and cellular apoptosis (Ni et al., 2017). It also has been found that extensive mitochondrial fission and defective mitochondrial fusion contribute to kidney diseases. Maintaining the balance of mitochondrial fission and fusion can restore mitochondrial function and subsequently protect renal function (Jiang et al., 2020a; Tang et al., 2021).

#### 3.4.1 Sirtuin activators

Sirtuins, due to its deacetylating activity, also play significant roles in the regulation of mitochondrial dynamics. Sirtuin-1 can deacetylate PGC-1α. PGC-1α regulates DRP1 expression by enriching in the promoter region to downregulate DRP1 expression and further inhibits mitochondrial fission (Ding et al., 2018; Dong et al., 2022). Study also has found that Sirtuin-3 can promote mitochondrial fusion *via* deacetylating and activating OPA1 (Samant et al., 2014).

Almost natural Sirtuin activators mentioned above are able to regulate mitochondrial dynamics in kidney diseases. For instance, polydatin is able to stabilize mitochondrial morphology and alleviate podocyte apoptosis *via* suppressing the expression and activation of DRP1 in DKD (Ni et al., 2017). Moreover, polydatin treatment attenuates sepsis-induced irregular or swollen mitochondria in renal TECs (Gao et al., 2015). Resveratrol can alleviate kidney injury *via* regulating mitochondrial dynamics. Hui et al. found that resveratrol alleviates excessive mitochondrial fission and promotes mitochondrial fusion in TGF-β1-treated mesangial cells (Li et al., 2021b). Curcumin can also reverse mitochondrial fragmentation in renal TECs *via* inhibiting mitochondrial fission and promoting mitochondrial fusion in cisplatin-induced kidney injury (Ortega-Domínguez et al., 2017; Cai et al., 2022).

#### 3.4.2 Nrf2 activators

Studies have demonstrated that Nrf2-ARE signaling pathway also plays a core role in the regulation of mitochondrial dynamics. Nrf2 can bind to the AREs in proteasomal gene promoter, which plays important roles in the degradation of DRP1. Moreover, activated Nrf2 can also dock on the AREs in Mfn2 gene promoter to promote mitochondrial fusion (Chen, 2022). Furthermore, Nrf2 can also upregulate Sirtuin-3 expression through binding to AREs in Sirtuin-3 promoter (Hu et al., 2021). Consequently, Sirtuin-3 can promote mitochondrial fusion *via* activating OPA1 (Samant et al., 2014).

A majority of Nrf2 activators can also regulate mitochondrial dynamics *via* the inhibition of mitochondrial fission and promotion of mitochondrial fusion in kidney diseases. For example, sulforaphane regulates mitochondrial dynamics and stabilizes mitochondrial morphology *via* downregulation of DRP1 in maleic acid-induced nephropathy and Fanconi syndrome (Briones-Herrera et al., 2018; Briones-Herrera et al., 2020). Astragaloside IV can protect against mitochondrial dysfunction and delay the progression of DKD *via* downregulating the expression of DRP1. In addition, Astragaloside IV treatment also ameliorates urinary albumin excretion and kidney injury in db/db mice (Liu et al., 2017).

Hyperoside, a Nrf2 nuclear translocation activator extracted from the flowers of *Abelmoschus manihot* (*L.*) *Medic*, shows a great potency in anti-inflammatory, anti-oxidation and anti-apoptosis effects (Chen et al., 2017). Hyperoside can alleviate excessive mitochondrial fission and promotes mitochondrial fusion *via* the inhibition of DRP1 and upregulation of Mfn (Chen et al., 2017). Furthermore, hyperoside is able to promote mitochondrial fusion and stabilize mitochondrial morphology *via* OPA1 in renal TECs (Wu et al., 2019).

## 4 Conclusion and perspectives

Although human kidney diseases arise from complicated pathological mechanisms and different etiologies, mitochondrial dysfunction is no doubt plays an important role in the pathogenesis. As a result, to maintain mitochondrial function has already shown to be a prospect strategy for therapeutic value in various kidney diseases. Hence, more and more studies in recent years have focused on the strategy targeting mitochondria in kidney diseases (Granata et al., 2015; Rahman et al., 2022; Tovar-Palacio et al., 2022).

Natural products, the mixture or monomer from animals, plants and microorganisms, with various bioactive ingredients, are huge valuable sources for drug candidates development (Katz and Baltz, 2016; Mu et al., 2020; Liang et al., 2021). In this review, we generally summarized the feasible strategies for attenuating mitochondrial dysfunction and restoring their function, including promoting mitochondrial biogenetics and energetics, reducing mitochondrial ROS, enhancing mitophagy and regulating mitochondrial dynamics. We focused on various kinds of natural products regulating mitochondrial function, such as AMPK activators, Sirtuin activators, Nrf2 activators and other antioxidants. This review may provide a practical guidance for clinic to choose appropriate natural products, from a new prospect, to restore mitochondrial function.

## Glossary

Ac: acetyl
acetyl-CoA: acetyl-coenzyme A
AKI: acute kidney injury
AMBRA1: activating molecule in BECN1-regulated autophagy protein 1
AMPK: AMP-activated protein kinase
ARE: antioxidant response element
ATP: adenosine triphosphate
BAX: Bcl-2-associated X protein
BINP3: B-cell lymphoma 2/adenovirus E1B 19-kDa-interacting protein 3
BINP3L: BINP3-like
BUN: blood urea nitrogen
CAPE: caffeic acid phenethyl ester
CKD: chronic kidney diseases
CPT1: carnitine O-palmitoyltransferase 1
CPT2: carnitine O-palmitoyltransferase 2
Cytc: cytochrome c
DKD: diabetic kidney disease
DRP1: dynamin related protein 1
EGCG: epigallocatechin 3-gallate
ERRs: estrogen-related receptors
ESRD: end-stage renal diseases
ETC: electron transport chain
FAO: fatty acid β-oxidation
FIS1: fission 1 protein
FoxO3a: forkhead box class O 3a
FUNDC1: FUN14 domain-containing protein 1
GSSG: oxidized GSSH
GSH: glutathione
GSPE: grape seed proanthocyanidin extract
GPX: glutathione peroxidases
HIF-1α: hypoxia-inducible factor-1α
IMM: inner mitochondrial membrane
IRI: ischemia-reperfusion injury
Keap1: kelch-like erythroid cell-derived protein with CNC homology-associated protein 1
LKB1: liver kinase B1
MiD49: mitochondrial dynamics proteins of 49
MFF: mitochondrial fission factor
Mfn1: mitofusin 1
mPTP: mitochondrial permeability transition pore
MQC: Mitochondrial quality control
**NAD** ^**+**^: nicotinamide adenine dinucleotide
Nrf2: nuclear factor-erythroid-derived 2-like 2
NRF: nuclear respiratory factors
OMM: outer mitochondrial membrane
OPA1: optic atrophy 1
OXPHOS: oxidative phosphorylation
Parkin: parkin RBR E3 ubiquitin-protein ligase
PA-S14: piericidin analogue S14
PINK1: PTEN-induced kinase 1
PGC-1α: PPARγ coactivator-1α
PPARα: peroxisome proliferator–activated receptor α
PNS: panax notoginseng saponins
PPARγ: peroxisome proliferator-activated receptor-γ
ROS: reactive oxygen species
SIRT1: sirtuin-1
SIRT3: sirtuin-3
SLC25A20: carnitine–acylcarnitine translocase
Scr: serum creatinine
SOD: superoxide dismutase
TCA: tricarboxylic acid
TECs: tubular epithelial cells
TFAM: mitochondrial transcription factor A
Ub: ubiquitin
UIRI: unilateral ischemia-reperfusion injury
UUO: unilateral ureteral obstruction.
